# Independent prognostic value of coronary artery calcium score and coronary computed tomography angiography in an outpatient cohort of low to intermediate risk chest pain patients

**DOI:** 10.1007/s12471-016-0819-5

**Published:** 2016-02-15

**Authors:** M. J. Bom, P. M. Van der Zee, F. M. Van der Zant, R. J. J. Knol, J. H. Cornel

**Affiliations:** Department of Cardiology, Medical Centre Alkmaar, Alkmaar, The Netherlands; Department of Nuclear Medicine, Medical Centre Alkmaar, Alkmaar, The Netherlands

**Keywords:** CCTA, Coronary computed tomography angiography, Prognosis, CACS, Coronary artery calcium, MACE

## Abstract

**Background:**

Limited studies report on the additional prognostic value of coronary computed tomography angiography (CCTA) and the coronary artery calcium score (CACS).

**Methods:**

For a median of 637 days, 1551 outpatients with chest pain, without known coronary artery disease (CAD) and low or intermediate pre-test probability of CAD, were followed for major adverse cardiac events (MACE), defined as death, myocardial infarction or late revascularisation. Cox proportional hazard regression was used to evaluate the independent prognostic value of CCTA and CACS.

**Results:**

MACE occurred in 23 patients (1.5 %): death (3, 0.2 %), myocardial infarction (4, 0.3 %) and late revascularisation (16, 1.3 %). Multivariate analysis showed an independent prognostic value of CCTA (*p* < 0.001), CACS of 100–400 (*p* = 0.035) and CACS of > 400 (*p* = 0.021). CCTA showed obstructive CAD in 3.1 % of patients with CACS = 0. No events occurred in patients with CACS = 0 without obstructive CAD at CCTA, whereas 2/23 patients (9 %) with CACS = 0 with obstructive CAD had a MACE.

**Conclusions:**

Our study shows that both CCTA and higher CACS categories have independent prognostic value in chest pain patients with low to intermediate pre-test probability of obstructive CAD, in which CCTA is appropriate. Furthermore a non-negligible amount of patients with CACS = 0 have obstructive CAD at CCTA. CCTA can be used in these patients to identify those at risk for MACE.

## Introduction

Quantification of coronary artery calcium by computed tomography represents a reliable estimate of atherosclerotic plaque burden and the prognostic value of the coronary artery calcium score (CACS) is well established [1, 2]. Recently, coronary computed tomographic angiography (CCTA) has emerged as an important imaging tool to detect the presence and extent of coronary artery disease (CAD) [3, 4]. Several large prospective trials have demonstrated the prognostic value of CCTA, with a high negative predictive value for the occurrence of major adverse cardiac events (MACE) [5–11]. Overestimation of the severity of CAD in patients with a high pre-test probability is, however, a known limitation of CCTA [12, 13]. Thus, the appropriate use criteria advise to only use CCTA in patients with low or intermediate pre-test probability [14]. The aim of this study was therefore to evaluate the independent prognostic value of CCTA and CACS in a routine clinical cohort of symptomatic patients with low or intermediate pre-test probability, in which CCTA is appropriate.

## Methods

### Population

From 13 December 2011 to 26 August 2014 all patients with chest pain with low or intermediate pre-test probability of CAD, referred for CCTA from the outpatient clinic after a diagnostic work up, were prospectively included. None of the patients had a prior history of CAD. The pre-test probability was calculated using the Duke Clinical Score [15]. A pre-test probability < 15 % was defined as low and 15–85 % was defined as intermediate, according to the ESC guidelines [3].

Baseline characteristics including age, gender, and cardiovascular risk factors were prospectively entered in the database. All patients gave written informed consent for usage of their data.

### Follow-up

Patients were followed for MACE, defined as all-cause mortality, myocardial infarction or revascularisation (either CABG or PCI). A 60-day landmark was used to differentiate between CCTA-driven invasive coronary angiography and late revascularisation, which is considered to be indicative for the prognostic value of CCTA. Patients with referral for invasive coronary angiography in the outpatient setting within 60 days after CCTA and subsequent revascularisation were considered to be CCTA-driven and not MACE. All other revascularisations within follow-up were considered to be MACE. Information on myocardial infarction and revascularisation was obtained from the electronic medical records. Information on mortality was obtained from the municipal personal records database.

### CCTA preparation, acquisition and analysis

Patient preparation, image acquisition, and image analysis were performed as previously described [16] and as briefly described below.

All scans were performed with a 2 × 64–slice flying focal spot, effectively 2 × 128–slice (Somatom Definition Flash; Siemens Medical Systems, Erlangen, Germany) and were evaluated by a Certification Board of Cardiovascular Computed Tomography accredited nuclear medicine physician and a cardiologist experienced in the interpretation of CCTA in consensus. In case of disagreement a third opinion was decisive.

### Radiation dose

The radiation dose delivered was generated automatically by the scanner software and represented as dose length product. The effective dose was calculated by multiplying the dose length product with the k-factor of 0.014 mSv × (mGy × cm)^−1^, which is generally used in cardiac CT studies.

### Definition of CAD

Obstructive CAD was defined as a lumen stenosis in any of the large vessels of > 50 %, either the left main artery, left anterior descending artery, circumflex artery or right coronary artery. Normal coronary arteries were defined as CACS = 0 and no coronary plaques. Non-obstructive CAD was defined as CACS > 0 and/or any plaque that did not meet the criteria for obstructive CAD.

### Statistical analysis

Statistical analysis was performed using SPSS software, version 22.0.0 (SPSS Inc, Chicago, Illinois). Continuous variables are presented as mean ± SD and categorical variables as frequencies with percentages. Continuous variables were tested for normal distribution.

Kaplan-Meier analysis was used to assess MACE-free survival stratified by CACS and CCTA results. Univariate and subsequent multivariate Cox proportional hazard regression were used to evaluate the independent prognostic value of CACS and CCTA beyond clinical risk factors. The multivariate Cox regression was done in stepwise fashion according to the backwards approach, with *p* < 0.10 as threshold for removal of variables. Clinical risk factors included in the univariate analysis were male gender, age, diabetes, smoking, hyperlipidaemia, hypertension, and family history of CAD. Selection of variables for entry in the multivariable Cox proportional hazard regression was based on univariate analysis with a threshold of *p* < 0.10.

## Results

### Population

A total of 1560 patients were initially included in the database for follow-up. Follow-up could be obtained in 99.4 % of the patients. Nine patients emigrated to a foreign country and were lost to follow-up. The total number of studied patients was 1551. An overview of the baseline characteristics is shown in Table 1.

**Table 1:** Baseline characteristics

Variable	Total (*n* = 1551)
**Demographics**	
Age	58.0 ± 10.2
Women	968 (62.4 %)
Body mass index	26.6 ± 4.5
Diabetes	121 (7.8 %)
Hba1c (*n* = 53)^a^	6.8 ± 1.6
Hypertension	464 (29.9 %)
Hyperlipidaemia	393 (25.3 %)
Family history of CAD^b^	731 (47.2 %)
Smoking	277 (17.9 %)
eGFR < 60	44 (2.8 %)
Duke clinical score	
– Low (< 15 %)	527 (34.0 %)
– Low-intermediate (15–50 %)	760 (49.0 %)
– High-intermediate (50–85 %)	264 (17.0 %)
**Baseline medication use**	
Aspirin	499 (32.2 %)
Statin	531 (34.2 %)
Beta-blocker	604 (38.9 %)
ACE-i/ARB	390 (25.1 %)
Calcium channel blocker	96 (6.2 %)
Nitrate	49 (3.2 %)
Acenocoumarol	38 (2.5 %)

*eGFR* estimated glomerular filtration rate, *ACE-I/ARB* ACE-inhibitor/angiotensin receptor blocker.

^a^Hba1C was documented in only 53 patients.

^b^In 2 patients family history of CAD was missing.

### CCTA data

CACS, CCTA results and radiation dose are summarised in Table 2. Two patients with left main CAD on CCTA also had three-vessel disease.

**Table 2:** CCTA data

Variable	Total (*n* = 1551)
**Coronary artery calcium score**	
0	739 (47.6 %)
0.1–100	498 (32.1 %)
100–400	215 (13.9 %)
> 400	99 (6.5 %)
**CCTA results**	
Normal coronary arteries	654 (42.2 %)
Non-obstructive CAD	683 (44.0 %)
Obstructive CAD (> 50 %)	214 (13.8 %)
1-vessel	164 (10.6 %)
2-vessel	34 (2.2 %)
3-vessel	13 (0.8 %)
Left main	4 (0.3 %)
High-risk lesions^a^	48 (3.1 %)
**Radiation dose**	
All patients (*n* = 1551)	2.4 ± 2.0 mSv
High-pitch FLASH scans (*n* = 1130)	1.6 ± 0.7 mSv
Prospectively triggered scans (*n* = 386)	4.5 ± 2.5 mSv
Retrospectively triggered scans (*n* = 35)	6.5 ± 2.7 mSv

*CCTA* coronary computed tomography angiography, *CAD* coronary artery disease, *PTP* pre-test probability.

^a^high-risk lesions were defined as left main, three-vessel and/or proximal left anterior descending disease.

### Follow-up

The cohort was followed for a median of 637 days. MACE occurred in 23 patients during follow-up. Three (0.2 %) patients died, 4 (0.3 %) patients had a myocardial infarction, and 20 (1.3 %) patients had non-CCTA-driven revascularisations. Four patients (0.3 %) were acute revascularisations in myocardial infarction and 16 (1.0 %) were late revascularisations, referred beyond 60 days after CCTA because of ongoing symptoms.

**Fig. 1:**
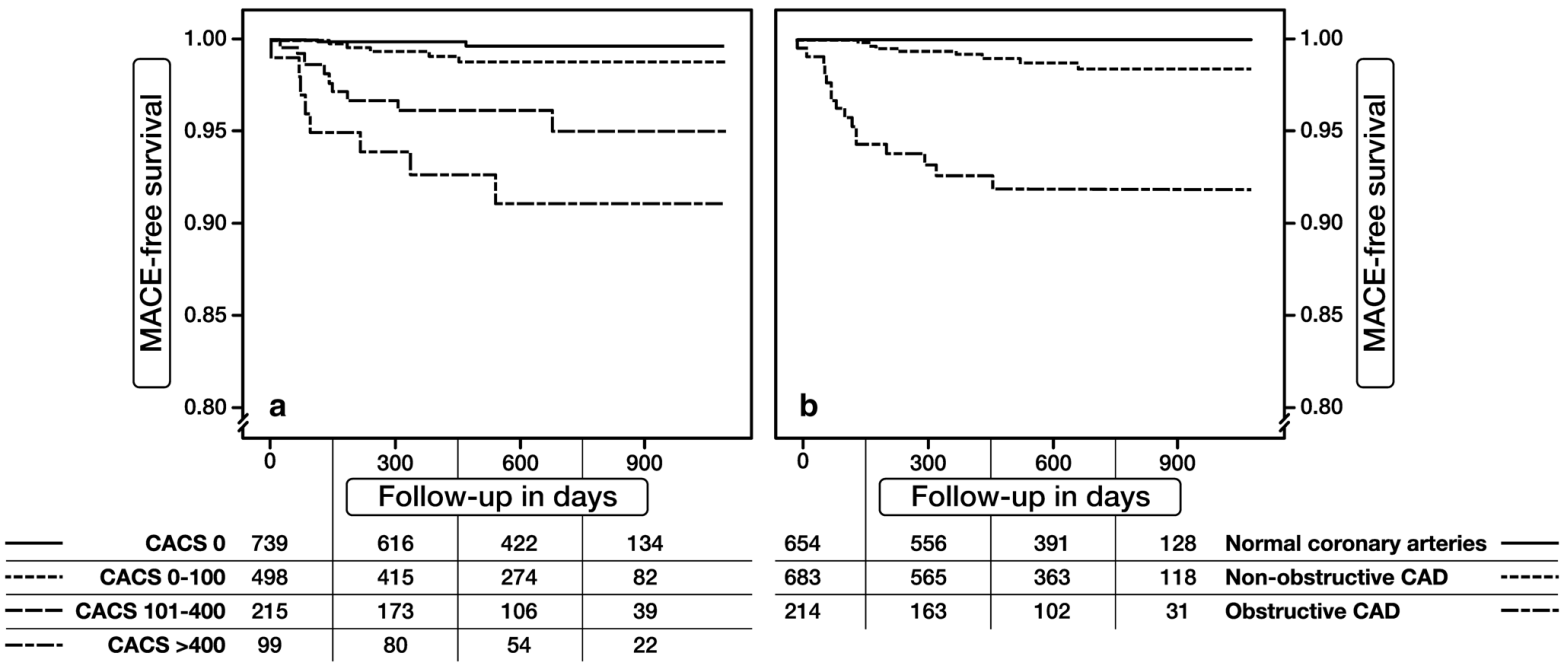
Kaplan-Meier curves of MACE-free survival stratified by CACS (**a**) and CCTA results (**b**)

Figure 1 shows the MACE-free survival estimates stratified by CACS and CCTA. Both increase in CACS and presence of obstructive CAD at CCTA were associated with decreased MACE-free survival (log-rank *p*-value < 0.01).

MACE occurred in no patients with normal coronary arteries at CCTA, in 1.0 % of patients with non-obstructive CAD at CCTA and in 7.5 % of patients with obstructive CAD at CCTA. During follow-up, MACE occurred in 0.3 % of patients with CACS = 0, in 1.0 % of patients with CACS 1–100, in 4.2 % with CACS 101–400 and in 7.1 % with CACS > 400.

Of all 739 patients with CACS = 0, 23 (3.1 %) had obstructive CAD at CCTA and 62 (8.4 %) had non-obstructive CAD at CCTA. No events occurred in patients with CACS = 0 and no obstructive CAD at CCTA, whereas 2/23 patients (9 %) with CACS = 0 and obstructive CAD at CCTA had a MACE during follow-up: late revascularisation in both cases. MACE-free survival was significantly worse in CACS 0 patients with obstructive CAD, log-rank p-value < 0.001.

### Independent prognostic value of CACS and CCTA

The evaluation of the predictive value of CACS and CCTA by univariate and multivariate Cox proportional hazard regression is shown in Table 3. By univariate analysis, increased risk of MACE was observed with increasing CACS (p-value < 0.001). Furthermore, obstructive CAD at CCTA was associated with a 13-fold increase in unadjusted risk of MACE. Of all clinical risk factors, male gender and hypertension had a p-value of < 0.10 and were subsequently included in the multivariate analysis.

**Table 3:** Univariate and multivariate Cox regression of risk factors, CACS and CCTA

Variable	HR	95 % CI	*P*-value
**Univariate analysis**			
Male gender	2.18	0.96–4.97	0.064^a^
Age	1.02	0.98–1.07	0.26
Diabetes mellitus	1.15	0.55–2.43	0.71
Hypertension	2.31	1.02–5.24	0.045^a^
Family history	1.76	0.76–4.07	0.19
Smoking	0.71	0.21–2.40	0.58
Hyperlipidaemia	1.97	0.85–4.55	0.11
CACS			
CACS = 0	Reference	Reference	-
CACS 0–100	3.71	0.72–19.12	0.12
CACS 100–400	15.82	3.42–72.23	< 0.001^a^
CACS > 400	26.81	5.57–129.09	< 0.001^a^
Obstructive CAD at CCTA	15.28	6.28–37.14	< 0.001^a^
**Multivariate analysis**			
Obstructive CAD at CCTA	7.03	2.57–19.22	< 0.001
CACS			
CACS = 0	Reference	Reference	-
CACS 0–100	2.46	0.46–13.14	0.29
CACS 100–400	5.97	1.14–31.31	0.035
CACS > 400	7.72	1.37–43.55	0.021

*CACS* coronary artery calcium score, *CCTA* coronary computed tomography angiography, *HR* hazard ratio

^a^variables with *p* < 0.10 on univariate analysis

In the multivariate model, obstructive disease on CCTA was independently associated with increased risk of MACE (*p* < 0.001). Furthermore, CACS of 100–400 (*p* = 0.035) and CACS of > 400 (0.021) were independent predictors for the occurrence of MACE, whereas a CACS of 0–100 (*p* = 0.29) had no independent predictive value for the occurrence of MACE.

## Discussion

Our study shows that in a routine clinical cohort of patients referred from the outpatient clinic with chest pain with low to intermediate pre-test probability of obstructive CAD, the prognosis of a CACS of 0 is excellent, with MACE during follow-up in only 0.3 % of patients. However, a non-negligible amount of patients with CACS = 0 had obstructive CAD (3 %). Presence of CAD in patients with CACS = 0 was associated with worse prognosis.

Furthermore, our study shows that although overall prognosis in this clinical cohort was excellent, presence of obstructive CAD on CCTA had an independent prognostic value. CACS of 0–100 did not show independent prognostic value of CCTA; however CACS 100–400 and > 400 did show independent prognostic value.

### Prognostic value of CACS and CCTA

The prognostic value of CACS has been previously demonstrated in large meta-analyses [1, 2]. A recently published large multicentre international cohort by Al-Mallah et al. reported a gradual increase in event rates in patients with CACS = 0, CACS 1–100, 100–399 and > 400, with comparable event rates to our study [10].

Multiple studies have previously reported on the prognostic value of CCTA for the occurrence of adverse events [6–11]. Our study results are in line with a recently published meta-analysis of 41,960 patients which reported that normal coronary arteries at CCTA are associated with a very low annual event rate of < 0.5 % and that obstructive CAD at CCTA is associated with higher event rates of 12.5 % [17]. The slightly lower event rate in patients with obstructive CAD at CCTA in our study might be explained by the fact that no patients with a high pre-test probability were included.

### CACS of 0

Our study, in accordance with prior studies, showed an excellent prognosis of symptomatic patients with CACS = 0, with an event rate < 1 % within 2 years of follow-up [10, 18–20]. Because of higher costs, the need to administer intravenous contrast and the higher radiation burden of CCTA, it remains the subject of discussion whether one should proceed with CCTA after CACS = 0 in all patients, given the excellent prognosis of these patients. The ESC guidelines on stable CAD do not recommend the use of CACS to identify individuals at risk [3]. However, a recent update of the American College of Cardiology and the American Heart Association stated that the exclusion of coronary calcium by CACS may be reasonable before considering further testing in symptomatic patients with low to intermediate pre-test probability [21]. Our study confirms findings of recent studies that in symptomatic patients with CACS = 0, a non-negligible 1.4–4.5 % have evidence of obstructive CAD at CCTA [10, 18, 19]. No events occurred during follow-up in patients with CACS = 0 and no obstructive CAD at CCTA in our study and obstructive CAD at CCTA was significantly associated with a worse prognosis. CCTA was thus able to accurately identify those with CACS = 0 at risk for future events. Performing CCTA after CACS = 0 may still be advisable in symptomatic patients with low to intermediate pre-test probability to identify those at risk for future events.

### Independent prognostic value of CCTA

Several studies have been published on the independent prognostic value of CCTA to CACS. Cho et al. reported that in 7590 studied asymptomatic patients, CCTA did not have prognostic value independent of CACS [22]. Other studies have shown that in predominantly symptomatic patients, CCTA has added prognostic value to CACS and clinical risk factors [6, 10, 23]. These studies, however, differ in study populations from our study. Hadamitzky et al. reported on the added prognostic value of CCTA to CACS in a population of both symptomatic and asymptomatic patients [23]. The single-centre study by Hou et al. included a substantial number of asymptomatic patients and patients with high pre-test probability [6]. Both Al-Mallah et al. and Van Werkhoven et al. studied solely symptomatic patients; 10 % of their population, however, had a high pre-test probability [8, 10]. The appropriate use criteria advise to only use CCTA with a low or intermediate pre-test probability [14]. While our findings are mainly of a confirmatory nature, our study does provide some additional information about the independent prognostic value of CCTA to CACS in a Dutch routine clinical cohort of symptomatic patients, in which CCTA is appropriate [14].

### Independent prognostic value of CACS

Data on the independent prognostic value of CACS in the CCTA era are limited [24–26]. Chaikriangkrai et al. recently reported independent prognostic value of both CCTA and CACS, with prognostic value across all categories of CACS [24]. In our study CACS had independent prognostic value, although only in the categories CACS 100–400 and > 400, whereas CACS 0–100 did not have prognostic value. This may be partly explained by the relatively low event rate and the lower risk population compared with the study population of Chaikriangkrai et al. The independent prognostic power of higher categories of CACS together with the fact that severe coronary calcification is associated with decreased diagnostic accuracy of CCTA, [27] support the use of CACS with CCTA as compared with CCTA only.

### Clinical implications

The recently published PROMISE trial investigated symptomatic patients with suspected CAD who require non-invasive testing and reported no difference in outcome between an initial strategy of CCTA and functional testing [28]. However, questions were raised about the safety of the CCTA strategy because of a relatively high radiation dose. The reported radiation dose in previous reports on the prognostic value of CCTA ranged from 3 to 18 mSv [5, 23, 28]. With the use of a high-resolution (2 × 128) scanner and predominantly a flash or prospective scanning protocol, we were able to perform a complete CCTA (calcium score and angiography) with an excellent prognostic value and a substantially lower radiation burden of 2.4 ± 2.0 mSv. Although the event rate was relatively low in our study, this further supports the use of an initial CCTA strategy in patients with low to intermediate pre-test probability of obstructive CAD.

## Study limitations

The single-centre design of our study allows the evaluation of prognosis in a ‘real world’ clinical setting. However, our results may not be applied to all outpatient chest pain populations, i.e. asymptomatic or high-risk patients.

As in previous studies, a 60-day landmark was used to differentiate between CCTA-driven invasive coronary angiography and long-term revascularisation, which is considered to be indicative for the prognostic value of CCTA [6]. Recently, several studies have reported that CCTA has a significant effect on downstream patient management [29, 30]. In our study 93 (6.0 %) patients underwent CCTA-guided revascularisation (referral for PCI/CABG within 60 days of CCTA). Since the aim of our study was to evaluate the prognostic value of CCTA in a routine clinical cohort, these patients were not excluded for analysis. However, outcome of the patients might have been confounded by CCTA-guided management changes.

## Conclusion

This study shows that both CCTA and CACS of 100–400 and > 400 have an independent prognostic value for the occurrence of MACE in a routine clinical cohort of patients presenting to the outpatient clinic with atypical chest pain with low to intermediate pre-test probability of obstructive CAD, in which CCTA is appropriate. Furthermore our study shows that a non-negligible amount of patients with CACS = 0 have obstructive CAD at CCTA. CCTA can be used in patients with CACS = 0 to identify those at risk for MACE.

### Funding

This work was not supported by any funding.

### Conflict of interests

None declared.
